# A Novel Spatiotemporal Classification of Eurasian Circulating African Swine Fever Virus Genotype II into Topotypes and Genetic Lineages

**DOI:** 10.3390/v18030346

**Published:** 2026-03-12

**Authors:** Roman Chernyshev, Alexey Igolkin, Sergey V. Shcherbinin, Alexander V. Sprygin

**Affiliations:** Federal Centre for Animal Health (ARRIAH), 600901 Vladimir, Russia; igolkin_as@arriah.ru (A.I.); sherbinin@arriah.ru (S.V.S.); sprygin@arriah.ru (A.V.S.)

**Keywords:** African swine fever, ASFV, genotype II, molecular epidemiology, Eurasia, SNP, phylogeny

## Abstract

African swine fever (ASF) has been a persistent threat to Eurasian pig populations since its emergence in 2007. The disease has become endemic in numerous countries, including Poland, Germany, Romania, Hungary, Italy, the Philippines, and several others. Epidemiological data reveals that over 99% of outbreaks are attributed to a highly virulent hemadsorbing virus belonging to genotype II. Traditional genotyping methods, primarily relying on the *B646L* gene, have faced significant limitations in providing a comprehensive understanding of virus dissemination patterns. Previous attempts to identify a universal marker for tracking virus spread through analysis of the CVR locus of the *B602L* gene and the *I73R/I329L* locus failed to produce a coherent picture of the virus’s geographical distribution across Eurasia. To address these challenges, a comprehensive study was conducted involving the analysis of 250 ASFV isolates/strains from 25 countries across Europe and Asia between 2007 and 2024. This research led to the development of a novel sub-genotyping algorithm for ASFV genotype II. The study identified four topotypes: «CAU1», «EU1», «EU2», and «ASIA1». Within these topotypes, 31 genetic lineages were detected, each characterized by specific single-nucleotide polymorphisms (SNPs). Based on the comparison of two methods of sub-genotyping Eurasian ASFVs—the classification by Gallardo C. et al. (2023) based on genetic variations of 6 loci, and the proposed classification into topotypes and genetic lineages using whole-genomes—it was established that the multigenic approach has insufficient resolution. At the same time, significant differences were observed at the level of whole-genomes. The creation of a new spatiotemporal classification has significant applications in international surveillance of ASF outbreaks, local disease monitoring, and investigation of new infection cases.

## 1. Introduction

**Figure 1:**
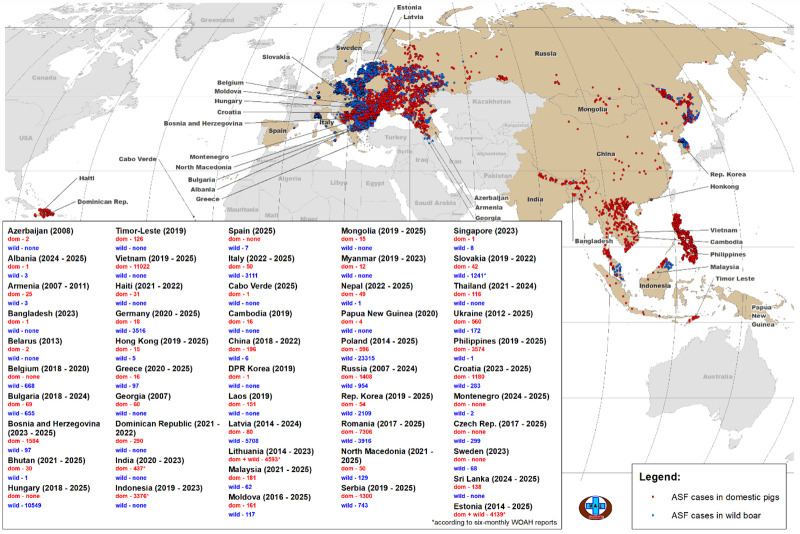
African Swine Fever epizootic situation in America, European and Asian countries, 2007–2025 (World Organization for Animal Health (WOAH) data as of 31 December 2025). Note: “dom”—the number of outbreaks among domestic pigs; “wild”—the number of wild boar outbreaks.

In the 21st century, within a relatively short period of time, African swine fever (ASF) has become a global problem. After the introduction of ASFV genotype II from Africa to Georgia (2007), the disease was notified in 47 European and Asian countries (2007–2025), as well as in the Caribbean region (Haiti and the Dominican Republic, 2021), taking on a panzootic character and causing serious economic damage to the pig-breeding and hunting industries (Figure 1) [1,2,3,4,5].

To date, many countries have reported ASF endemicity (Poland, Germany, Romania, Hungary, Italy, the Philippines, and others) [5,6,7,8,9]. The onset of a new panzootic marked the development of alternative approaches to national and international surveillance, control, and disease management [10]. One such approach was formed by molecular epidemiology methods [11].

The etiological agent of ASF is an enveloped virus with double-stranded DNA, the only representative of the genus *Asfivirus* and family *Asfarviridae*, belonging to the group of large nuclear–cytoplasmic DNA-containing viruses (*Nucleocytoviricota*) [12]. Its genome ranges from 170 to 194 kbp, contains 150–195 open reading frames (ORFs), and consists of a central conserved region (CCR) of 125 kbp and two variable terminal regions (VR) [13]. The identity of CCR between known ASFV sequences is ≥98.5% [14]. The VR includes predominantly multigene families (*MGF100*, *110*, *300*, *360*, *505*) [15]. The genome contains a large number of tandem inverted repeats and homopolymers, and it is helical due to the presence of terminal loops [16,17,18]. AT-rich, easily denatured DNA regions are characteristic [19]. The traditional target for ASFV genotyping is a 475 bp fragment of the C-terminal region of the *B646L* gene encoding the capsid protein vp72 [20].

It is worth noting that ASFV transmission cycles in Africa and Eurasia differ. In African countries, the sylvatic transmission cycle predominates, involving *Ornithodoros moubata* ticks and warthogs (*Phacochoerus africanus*); meanwhile, the Eurasian continent is characterized by the “domestic pig/wild boar” cycle [21]. Consequently, the frequency of genetic changes may vary. In Eurasia, ASFV exhibits a process of co-divergence (co-evolution with the host), resulting in a mutation rate comparable to that of poxviruses (5.72 × 10^−6^ substitutions/site/year) [22,23]. Domestic pigs (*Sus domesticus*) and wild European boars (*Sus scrofa*) are new components of the transmission chain, historically non-typical hosts for ASFV, which, due to the moderate rate of molecular evolution, results in a slowed attenuation of its virulent properties.

However, despite key differences in ASF epidemiological features between Africa and Eurasia over 18 years of circulating genotype II virus, non-hemadsorbing low-virulent variants were discovered in Latvia in wild boar (2017) and in China among domestic pigs (2020), causing atypical (chronic) disease progression [24,25]. These variants are characterized by deletions of genes encoding virulence factors (CD2v protein responsible for hemadsorption; *MGF 110-1L* gene, and others). However, the detection of atypical variants of genotype II virus is exceptional, while >99% of ASF outbreaks in Eurasia are caused by highly virulent hemadsorbing virus [26].

However, substitutions in ORFs and insertions of tandem repeats (TRs) in intergenic regions generally do not significantly alter the biological properties of the ASFV [27]. Nevertheless, their applied significance in spatiotemporal studies of virus circulation is substantial and continually updated [10].

The sub-genotyping method (division of isolates into additional taxa within a genotype) has long been used in the molecular genetic characterization of pathogens of many economically significant and highly dangerous animal diseases (classical swine fever, highly pathogenic avian influenza, Newcastle disease, foot-and-mouth disease, and others) [28,29,30,31]. In particular, foot-and-mouth disease virus within a specific serotype is divided into topotypes, genetic lineages, and sub-lineages in order to track the spread of the disease [31]. Due to the significant variability of the *B646L* gene (presence of all genotypes), the issue of ASFV sub-genotyping circulating in African countries was not relevant. Instead, additional loci for analysis in some studies included the central variable region (CVR) of the *B602L* gene and *E183L* [32,33,34,35,36,37]. However, the ASFV of genotype II exhibits vp72 conservation over 18 years of circulation in Eurasia. Therefore, it is necessary to search for alternative nucleotide regions in the genome of genotype II virus due to its widespread distribution.

Indeed, various genetic variants of the ASFV originating from the Georgia 2007/1 strain, described in European and Asian countries, are characterized by specific single-nucleotide polymorphisms (SNPs) and evolve within a constantly expanding range. This fact allowed Gallardo C. et al. (2023) to develop a sub-genotyping method identifying 24 clusters (genetic groups) in Europe [38]. However, such classification does not account for most variants characterized in Asia and is based on the use of only six genomic marker regions (CVR, *I73R/I329L*, *K145R*, *O174L*, *MGF 505-9R/10R*, and *I215L*). The large volume of information does not provide a complete understanding of how to universally sub-genotype new ASFV isolates. Moreover, marker selection is often inductive and not based on in-depth research of numerous whole-genome sequences, isolate phylogenomics, and epidemiological (historical) facts. Furthermore, several studies have already demonstrated the monophyletic origin of ASFVs genotype II in Eurasia [10,39]. Therefore, it is necessary to consider the epizootic in Eurasia (2007–present) as a discrete system and the Georgia 2007/1 strain as the root node.

A major obstacle in reconstructing the ASFV spread pattern is the lack of whole-genome sequences of isolates published in genetic databases before 2021. Currently, GenBank has significantly expanded with information about new isolates from both Europe (Italy, 2022–2023; Serbia, 2020–2022; Germany, 2020–2022; Russia, 2019–2021; Lithuania, 2018–2019) and Asia (China and Hong Kong, 2019–2024; Mongolia, 2019–2021; Russia, 2020–2022; South Korea, 2019–2021; Vietnam, 2019–2020; the Philippines, 2019–2023; India, 2020–2022; Papua New Guinea, 2020–2023; and others) [22,40,41,42,43,44,45,46,47,48,49]. As a result, differentiation of field isolates of ASFV genotype II registered in Eurasian countries based on complete genomes has become possible.

Thus, the aim of the study was to develop a sub-genotype classification in accordance with current data from whole-genome analysis of ASFV genotype II strains isolated in Eurasia between 2007 and 2024.

## 2. Materials and Methods

Sequences. For comparative analysis of studied isolates, genome sequences of ASFV genotype II strains and isolates from Eurasian territories were imported from the GenBank international database. Sequence selection for the study was carried out based on the principle of highest homology (99.99–99.87%) with the reference strain Georgia 2007/1 using the NCBI: Nucleotide BLAST online platform. As a result, 258 files (in FASTA format) were imported from the GenBank database, representing whole-genome annotated sequences of isolates and strains of ASFV belonging to genotype II and isolated from 26 Eurasian countries (2007–2024).

Alignment and primary trimming. The pool of ASFV whole-genome sequences was aligned using MAFFT (Galaxy Version 7.526 + galaxy2) with the ‘–auto’ flag, which automatically selects an appropriate MAFFT algorithm [50]. The alignment input parameters include a simple multiple sequence alignment (MSA) for all sequences, a scoring matrix type of Kimura PAM200, and the FFT-NS-2 strategy. Trimming was performed by removing sequences with unaligned 5′-ends in the left VR. Homopolymers and polyC/G tracts have been removed. After trimming, 8 isolates with large unaligned regions were excluded to avoid distortion of results. Thus, the analysis included 250 whole-genome sequences of ASFV genotype II from 25 countries. The list of studied isolates with their NCBI GenBank accession numbers and available epidemiological data is presented in Supplementary Table S1.

Phylogenetic Analysis. Prior to phylogeny inference, the obtained alignment was trimmed with trimAl v.1.5 rev0 build 27 May 2024 with default settings [51]. The best-fitting DNA substitution model TPM1uf + G4 was selected using ModelTest-NG [52]. The recommended DNA substitution model and the cleaned alignment were used as input for Randomized Accelerated Maximum Likelihood (RAxML) to construct the ML tree (with bootstrap of 1000 replicates) [53]. The resulting ML distances were visualized with iTOL [54].

Mapping. Molecular-epidemiological maps were created using QGIS v.3.44.7 software [55].

## 3. Results

**Figure 2:**
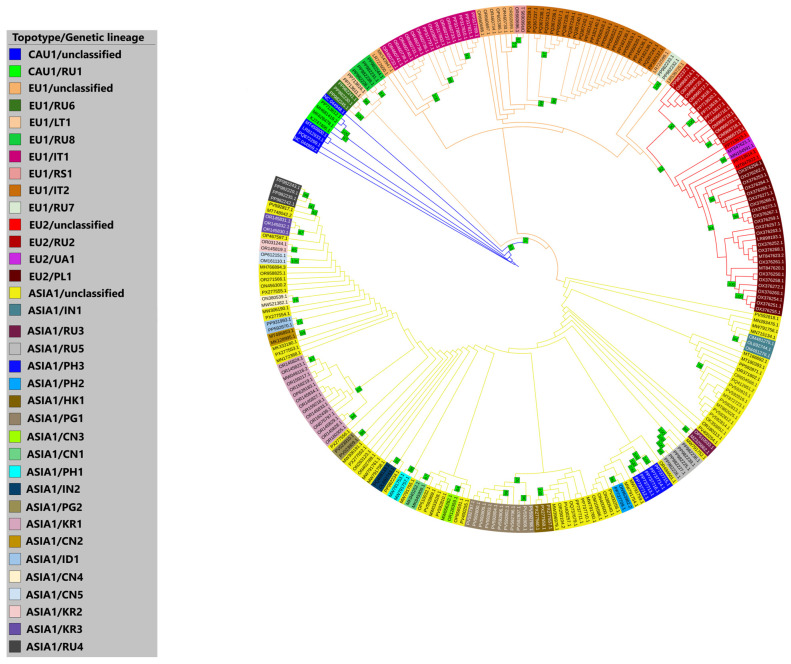
Circular cladogram constructed using the maximum likelihood method, showing the phylogenetic relationships of genotype II isolates collected in Eurasia. *Note: The tree is rooted relative to the Georgia 2007/1 strain. The branch color corresponds to the topotype, while the leaf color indicates the genetic lineage. Some clades with a bootstrap ≥ 65 are not designated as autonomous genetic lineages because specific SNPs for their differentiation were not subsequently found*.

A phylogenetic analysis conducted using the ML method visualized a cladogram reflecting the topology of daughter taxa divergent from the Georgia 2007/1 strain (Figure 2). The deductive approach used in interpreting the dendrogram allowed us to propose a new classification of ASFV genotype II circulating in Eurasia into topotypes and genetic lineages.

The classification concept of taxa is based on the phylogenetic-geographical affiliation of isolates to specific groups with a strictly differentiated set of single-nucleotide polymorphisms (SNPs). The identification of topotypes is based on the spatiotemporal factor, while genetic lineages are based on a high confidence index (bootstrap ranging from 65 to 100) when dividing isolates within a topotype.

Four topotypes were identified: «CAU1» (Caucasus 1), «EU1» (Europe 1), «EU2» (Europe 2), and «ASIA1», forming major clades divergent from the Georgia 2007/1 strain, designated as the root node. Isolates within each topotype possess at least one SNP characteristic of all representatives of this taxon.

All four topotypes contain internal branches forming genetic lineages. In the absence of a characteristic SNP or in cases of single occurrence (where a sister clade consists of only one terminal node), the taxon is designated as «unclassified». Ultimately, 31 lineages were classified (Figure 2, Table 1). When typing a new isolate, genetic variants characteristic of the topotype must strictly adhere to the guidelines, but at least one specific SNP is sufficient for assigning the isolate to a classified genetic lineage. This approach is based on the principle of common ancestry, regardless of the time and place of ASFV isolation.

The names of topotypes indicate the top-level domains of specific territories where the virus predominantly circulates. The names of genetic lineages take into account the country of the first detection. Since the virus of certain taxa can be registered in countries other than its first detection, only top-level domain designations are present in the names of genetic lineages. This is done to avoid direct association with a country, as any names in this case are formal. The initial detection of the virus in a particular country does not indicate the emergence of specific SNPs within that territory, nor does it imply that ASFV is confined to the detection cluster. The numbers in names of topotypes and genetic lineages are needed to organize the chronology of virus variants emergence in Eurasia.

The earliest and most homologous genovariants to the Georgia 2007/1 strain are united into the «CAU1» topotype, designated in blue. Within it, the «RU1» lineage is identified, which includes strains isolated in Russia (2013), Lithuania (2014–2019), and Poland (2015).

The divergence of genetic variants registered in Europe and Asia into the «EU1» topotype, designated in orange, is clearly reflected. The «EU1» topotype forms two clades marked in red (the «EU2» topotype) and yellow (the «ASIA1» topotype).

Representatives of the «EU1» topotype belong to the I267L-II, MGF 110-7L-I, MGF 505-5R-I, K145R-I, and MGF 360-10L-I variants and are classified into seven genetic lineages: «RS1»—isolates from Serbia (2019–2021); «RU6»—isolates from the Belgorod, Perm, and Sverdlovsk regions of Russia, identified in 2021; «LT1»—isolates identified among wild boars in Lithuania (2018–2019); «RU7»—a genetic variant first identified in 2021 during an outbreak at a large pig farm in the Bryansk region of Russia; «RU8»—registered in central (Pskov and Belgorod) and eastern (Amur and Khabarovsk) regions of Russia; «IT1»—constitutes a unique molecular-epidemiological cluster on the border of Piedmont (Alessandria) and Liguria (Genoa) in Italy; «IT2»—isolates identified in 2023 in the cluster in the Calabria region of Italy (Reggio di Calabria).

The most studied polymorphism revealed a genetic variant circulating in Ukraine, Poland, Russia, Germany, and Lithuania. Homologous isolates are united into the «EU2» topotype, characterized by I267L-II, MGF 110-7L-II, MGF 505-5R-II, K145R-II, and MGF 360-10L-I variants. Within the «EU2» topotype, at least three genetic lineages can be identified: «UA1»—first registered in the Kyiv region of Ukraine (2016), later noted in Poland (2018); «RU2»—a previously unique variant for the Kaliningrad region of Russia with an additional SNP in the *K145R* gene, which is also detected in Lithuania (2019) and sporadically in East Russia (2021); «PL1»—comprises isolates first identified in Poland (2017–2019) and all known isolates from Germany (2020–2021), both in Brandenburg and Saxony federal states.

All isolates allocated to the «ASIA1» topotype have a «T -> C» transversion in the *MGF 360-10L* gene and are registered in Asian countries, with the exception of one strain from Hungary (ASFV HU 2018), which is the probable ancestral form for all Asian isolates. All representatives of this topotype are characterized by the following variants: I267L-II, MGF 110-7L-I, MGF 505-5R-I, K145R-I, and MGF 360-10L-II. Twenty genetic lineages are identified within the «ASIA1» topotype: «IN1»—unique to ASFV isolated in India; «IN2»—first registered in the Indian state of Assam (2020) and later noted in Nepal; «ID1»—characteristic of two isolates identified in 2019–2020 on Indonesian islands; «RU3», «RU4», and «RU5»—lineages currently registered only in limited territories of eastern regions of Russia; «KR1», «KR2», and «KR3»—three lineages noted only in South Korea; «PH1», «PH2» and «PH3»—three taxa identified in the Philippines, each constituting an ASF outbreak cluster; «CN1»—ASFV strains registered at the beginning of the epizootic in northern Chinese provinces (Jilin in 2018 and Inner Mongolia in 2019); «CN2»—characteristic of Anhui and Guangdong provinces of China; «CN3»—comprises ASFV isolates from northern and central China; «CN4» and «CN5»—registered in Hubei province of China in 2020–2021; «HK1»—isolates identified in Hong Kong in 2023–2024; «PG1» and «PG2»—unique lineages identified in Papua New Guinea in 2020–2021 and 2023, respectively.

For the systematic recording of sub-genotypes in the future, we propose using the following format: «II/topotype/genetic lineage», such as «II/CAU1/unclassified».

**Figure 3:**
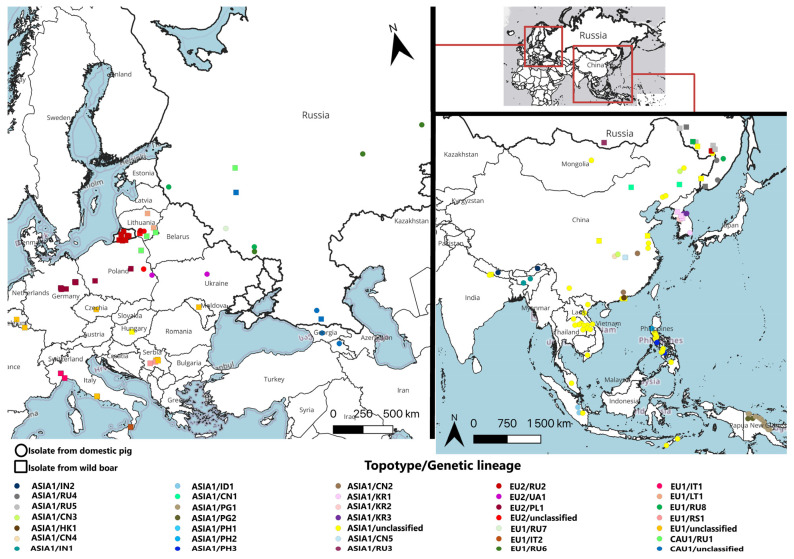
Occurrence of African swine fever virus genotype II topotypes/genetic lineages in Europe and Asia.

The occurrence of genetic lineages is shown on the map (Figure 3).

With the exception of Lithuania, Poland, and Russia, the circulation of ASFV genotype II variants within a single topotype was detected for all administrative regions of the Eurasian continent countries. In Lithuania, three topotypes were registered («CAU1», «EU1», and «EU2»), while in Poland, two topotypes were identified («CAU1» and «EU2»). In Russia, all four topotypes were detected. Thus, the geographical affiliation of ASFV genotype II topotypes to most specific regions of European and Asian countries has been demonstrated.

The next step involved comparing our proposed classification, based on whole-genome sequences of genotype II ASFVs isolated in Eurasia, with the data on genetic groups according to the distribution of genetic variants across 6 loci, as proposed by Gallardo C. et al. [38] (Table 2).

As shown in Table 2, no correlation has been established between the divergence of ASFV genotype II in Eurasia based on whole genomes and 6 marker regions. In most cases, genetic variants belonging to topotypes «CAU1», «EU1», and «ASIA1» are part of genetic group 3, whereas genetic lineages of the «EU2» topotype belong to genetic group 7.

As a result of whole-genome phylogeny, 31 genetic lineages of ASFV genotype II in Eurasia have been classified, while analysis of 6 loci corresponds to 4 classified and 4 unclassified genetic groups. The low resolution of locus-based differentiation underscores the primary importance of conducting whole-genome analysis.

## 4. Discussion

Phylogenomics is the science studying the evolution of organisms’ genomes. The spatiotemporal approach in this field can help reconstruct the epizootic pattern of ASF spread. The main task is to determine the phylogenetic relationship of isolates and identify genetic variants of the ASFV circulating in a specific territory [10].

Over more than 18 years of the epizootic caused by ASFV genotype II, originating from the Georgia 2007/1 strain, numerous substitutions (SNPs) have emerged in the large genome (189.5 kbp) during the virus spread in the animal population, leading to the divergence of the original (parental) strain into many genetic variants (Table 1). Molecular evolution in this case did not significantly affect the change in the biological properties of the virus but allowed differentiation of field isolates based on their geographical (epidemiological) characteristics.

The search for a universal marker of spread (genome region), reflecting the topology of the phylogenetic tree using complete genomes of ASFV genotype II, was unsuccessful [38]. It is important to note that attempts to cluster isolates by the CVR locus of the *B602L* gene and *I73R/I329L* only provided scattered data and did not demonstrate a holistic picture of virus spread across the Eurasian continent [56]. Additionally, the use of indels in the ASFV genome (TRs, deletions *EP153R/EP402R*, etc.) also contradicted the spatiotemporal pattern due to their autonomous emergence in epidemiologically unrelated areas [57,58,59,60].

Based on the comparison of genetic variants of 6 loci and whole genomes of ASFVs, marker homogeneity was established in most cases, while differences at the level of whole genomes were significant (Table 2). This once again emphasizes the insufficient resolution of locus-based analysis in tracking the spread of genotype II ASFVs and the inconsistency of phylogenetic tree topologies based on loci and whole genomes.

Due to these limitations, this study proposes a tool for reliable sub-genotyping of isolates within genotype II using only whole-genome analysis to understand the global spread of ASFV. The study of scientific literature and genetic databases allowed developing a new classification of ASFV genotype II circulating in Eurasia. Prerequisites for such generalization existed earlier and were mentioned in search works of scientific groups from many countries, but isolates were not clearly classified until this study [22,26,39,61]. The proposed classification is fully based on phylogenetic relationships (Figure 2) and relies on identifying known SNPs (Table 1). The nomenclatural division into topotypes and genetic lineages reflects the spread of isolate pools in various territories of Eurasia (Figure 3).

The system of randomized SNPs in the genome, arising randomly during virus evolution, is the initial basis for clustering. It aligns with the results of phylogeographic (Bayesian) analysis performed by Gambaro F. et al. and Rossi G. et al. in 2025, which largely clarified typical misconceptions about the spread of ASFV genotype II [22,26]. The history of spread is clearly illustrated in the phylogenetic tree (Figure 2). The initial genetic variant—topotype «CAU1»—was introduced from Africa to Georgia in 2007 and was subsequently found in Russia until at least 2019 [41]. In 2014–2015, topotype «CAU1» spread to Europe, where it later gave rise to a variant—topotype «EU1». This variant was subsequently registered in Russia, Ukraine, Moldova, the Czech Republic, Belgium, Serbia, and Italy [40,41,62,63,64,65]. At least by 2016, an ASFV diverged from «EU1», named topotype «EU2». It was first registered in Ukraine and later detected in Poland, Russia, Lithuania, and Germany [22,38,62,66,67,68]. «EU2» is likely also present in Romania, as it exhibits the K145R-II genetic variant based on the marker locus [38]. However, no whole-genome of ASFV from Romania have yet been deposited in GenBank, so conclusions are premature. During 2017–2018, another genetic variant with a single specific substitution in the *MGF 360-10L* gene diverged from topotype «EU1». This event occurred in Europe, but it is difficult to determine exactly which country. Phylogenetically, the Hungarian isolate (HU 2018) is the only European virus with this SNP published in GenBank [69]. This genetic variant entered China in 2018 and spread unprecedentedly throughout Asia [10,70]. Since the marker substitution has been identified, it is reasonable to designate this variant as topotype «ASIA1», although it is otherwise very similar to «EU1». Of course, within each of the four topotypes, the ASFV evolved and divided into sub-variants with specific SNPs. These were designated as genetic lineages in nomenclature. Thus, our proposed classification aligns with the objectives of the study and the historical context of ASFV spread in Eurasia.

The proposed method has both advantages and disadvantages. An undeniable advantage is the lack of discreteness: the developed classification is not finite; it only outlines the principle of isolate division. Due to the continuous epizootic and subsequent molecular evolution of ASFV in Eurasia, the divergence of genotype II virus will only increase, inevitably forming new genetic lineages and possibly even topotypes, allowing for more precise molecular tracking. Moreover, there is a high prospect of isolating sub-lineages as nomenclatural units. For example, Figure 2 shows that within the genetic lineage «PL1», clades with high bootstrap indices can be further identified, including isolates from specific German lands (Brandenburg and Saxony). Several sub-lineages can also be identified within genetic lineages «IT1», «PH3», and «RU4». In any case, this is already a task for local studies. Additionally, this method can detect intragenotypic recombination. For example, several topotypes of the virus circulate in Lithuania, Poland, and Russia, making genetic recombination highly probable. The detection of specific SNPs characteristic of more than one topotype/genetic lineage suggests the possibility of genetic interactions within genotype II.

Additionally, when using the proposed method in routine laboratory practice, it may not always be necessary to construct phylogenetic trees. It will suffice to simply perform consensus alignment of the examined sample against the reference genome of the Georgia 2007/1 strain, and subsequently distribute it into a topotype/genetic lineage based on specific SNPs (Table 1). Of course, if an unclassified lineage is identified, phylogenetic analysis should be performed using a comprehensive set of sequences.

The main disadvantage of the proposed method is its relatively high cost. High-throughput sequencing of the 2nd and 3rd generation (Illumina, IonTorrent, BGI/MGI, PacBio, Oxford Nanopore, and others) requires significant expenses, while Sanger sequencing is unlikely to be applicable in this context due to the constant emergence of new (uncharacterized) SNPs and, consequently, new genetic lineages of ASFV. It is also necessary to prove the absence of circulation of ASFV topotype «ASIA1» in Europe, especially in Hungary. If it continues to circulate, it may distort the spread pattern and lead to false conclusions about the secondary introduction of ASFV into Europe from Asian countries.

It is also necessary to acknowledge that the number of whole-genome sequences of ASFVs isolated in Eurasia and published in GenBank is extremely small. Among approximately 80,000 ASF outbreaks on the continent, 250 genomes represent ~0.003%. It is also worth noting that the sampling effort of isolates from different countries is heterogeneous. For example, numerous whole-genome sequences of ASFV from Italy, Germany, or Papua New Guinea have been published, while there are none from Romania—despite the extremely high number of outbreaks in that country. The lack of transparency in molecular and epidemiological data from some countries also reduces the reliability of the results obtained. Due to these limitations, conclusions about the origin and spread of ASF outbreaks must be made with great caution, taking into account their hypothetical nature.

Thus, a new classification of sub-genotypes (4 topotypes and 31 genetic lineages) of ASFV within genotype II has been scientifically substantiated and proposed, which will significantly expand the surveillance capabilities in investigating ASF outbreaks in the future. Further publication of ASFV isolate genomes from Eurasia in databases will increase the genetic data array, leading to a deeper understanding of the disease spread pattern.

## Figures and Tables

**Table 1:** Guidelines for sub-genotyping of African swine fever virus isolates belonging to genotype II into topotypes and genetic lineages.

№	Topotype Name (Differentiating SNP for Topotype)	Genetic Lineage Name	Specific SNP for Lineage	Known Strains/Isolates Comprising the Genetic Lineage
1	**CAU1** I267L-I MGF 110-7L-I MGF 505-5R-I K145R-I MGF 360-10L-I	**RU1**	MGF 360-10L-III (A -> G: 27009); EP1242L-III (G -> A: 69509)	Kashino 04/13; ASFV/LT14/1490; SFV/POL/2015/Podlaskie; LTU/2019/00332G2S1
2	**EU1** I267L-II (T -> A: 170862) MGF 110-7L-I MGF 505-5R-I K145R-I MGF 360-10L-I	**RS1**	MGF 360-19Ra-II (C -> T: 186844)	DG 6314 19; DG 6511 21
3		**RU6**	F1055L-II (C -> T: 61821); B962L-II (T -> C: 95794); B318L-II (G -> A: 96624); B354L-II (G -> A: 101182); QP383R-II (C -> T: 161600)	ASFV/Belgorodskaya/2021/DP-1838; ASFV/Permskyi 2021/DP-9916; ASFV/Sverdlovskaya 2021/DP-9914
4		**LT1**	MGF 505-6R-III (A -> G: 40340); C962R-II (C -> T: 90558); H233R-II (G -> A: 155828)	LTU/2018/00696S3; LTU/2019/08805G2S1
5		**RU7**	MGF 505-10R-II (G -> A: 44576); MGF 360-15R-II (G -> A: 50655 + G -> A: 50667); F334L-II (C -> T: 57933); NP1450L-II (G -> C: 131463); Q706L-II (C -> G: 158805)	Bryanskaya_2021/DP-18; Bryanskaya_2021/DP-8823
6		**RU8**	MGF 360-18R-II (G -> A: 184569)	ASFV/Belgorodskaya 2021/DP-11869; ASFV/Pskovskaya 2021/DP-9008; ASFV/Amur 2022/WB-909; ASFV/Khabarovsk 2022/DP-1658
7		**IT1**	MGF 110-9L-II (G -> A: 13088); MGF 300-4L-II (G -> A: 23709); MGF 360-15R-III (G -> A: 50687); EP1242L-II (G -> A: 70320); NP1450L-III (T -> C: 134360); I196L-II (A -> G: 176090)	2157/AL/2022 Italy; 50665.8 2167/AL/2022 Italy; 8549_2284/AL/2023_Ita; 22700_2598/AL/2023_Ita; 22700_2619/AL/2023_Ita; 22700_2628/AL/2023_Ita; 22700_2645/AL/2023_Ita; 22700_2646/AL/2023_Ita; 50665.1 2170/AL/2022 Italy; 2802/AL/2022 Italy; 47169.12 1495/GE/2022 Italy; 50665.12 2152/GE/2022 Italy; 47169.16 1499/GE/2022 Ita; 47169.14 1497/AL/2022 Italy; 50665.5 2168/AL/2022 Italy; 50665.4 2159/AL/2022 Italy
8		**IT2**	MGF 110-3L-II (C -> T: 8417)	55135_2735/RC/2023_Ita; 23324_2335/RC/2023_Ita; 55135_2741/RC/2023_Ita; 55135_2743/RC/2023_Ita; 26254_2321/RC/2023_Ita; 23249_2337/RC/2023_Ita; 23809_2342/RC/2023_Ita; 25787_2389/RC/2023_Ita; 55135_2732/RC/2023_Ita; 55135_2740/RC/2023_Ita; 55135_2737/RC/2023_Ita; 25791_2390/RC/2023_Ita; 55135_2736/RC/2023_Ita; 21896.3_2307/RC/2023_Ita; 55135_2742/RC/2023_Ita; 55135_2738/RC/2023_Ita; 23276_2329/RC/2023_Ita; 22489.4_2312/RC/2023_Ita; 23251_2316/RC/2023_Ita; 23287_2331/RC/2023_Ita; 23259_2323/RC/2023_Ita; 23317_2333/RC/2023_Ita; 55135_2734/RC/2023_Ita
9	**EU2** I267L-II MGF 110-7L-II (G -> A: 10668) MGF 505-5R-II (G -> A: 39306) K145R-II (C -> A: 66152) MGF 360-10L-I	**UA1**	MGF 360-16R-II (G -> A: 178478)	ASFV/Kyiv/2016/131; Pol18 28298 O111
10		**RU2**	K145R-III (C -> T: 66009)	ASFV/Kaliningrad 18/WB-12523; ASFV/Kaliningrad 17/WB-13869; ASFV/Kaliningrad 18/WB-12516; ASFV/Kaliningrad 18/WB-9766; ASFV/Kaliningrad 19/WB-10168; ASFV/Kaliningrad 18/WB-12524; ASFV/Kaliningrad 18/WB-9763; ASFV/Kaliningrad 18/WB-9734; ASFV/Kaliningrad 18/WB-9735; ASFV/Khabarovsk 2021/WB-3967; LTU/2019/09335GS2; LTU/2019/09790P1G1; LTU/2019/09074GSV11; LTU/2019/09074GSV1
11		**PL1**	K205R-II (G -> A: 64395)	Pol19 53050 C1959/19; Pol17 55892 C754; 2020ASP02894; 2021ASP01917; 2021ASP00484; 2020ASP02103; 2021ASP03251; 2021ASP03711; 2021ASP03658; 2021ASP01957; 2021ASP02148; 2021ASP02207; 2021ASP02665; 2021ASP03740; ASFV Germany 2020/1; 2021ASP00902; 2021ASP00921; 2021ASP03643; 2021ASP01919; 2021ASP00703; 2020ASP01832; 2020ASP02805; 2021ASP03384; 2021ASP03380; 2021ASP03144
12	**ASIA1** I267L-II MGF 110-7L-I MGF 505-5R-I K145R-I MGF 360-10L-II (T -> C: 26425)	**IN1**	MGF 505-4R-II (G -> A: 37442)	ABTCVSCK ASF001; ABTCVSCK ASF007; IND/AR/SD-61/2020
13		**RU3**	KP177R-II (G -> A: 4400); A137R-II (C -> T: 55804)	ASFV/Zabaykali/WB-5314/2020; ASFV/Zabaykali 20/DP-4905
14		**RU4**	CVR-XIII (C -> T: 102721); NP868R-II (A -> G: 136176)	ASFV/Amur 2022/WB-911; ASFV/Khabarovsk 2020/DP-11562; ASFV/Primorsky 2021/DP-9778; ASFV/Primorsky 2021/WB-9786
15		**RU5**	MGF 505-3R-II (A -> G: 36136); F778R-II (G -> A: 58721)	ASFV/Amur 2021/WB-10591; ASFV/Amur 2021/WB-10595; ASFV/Amur 2022/WB-905; ASFV/Khabarovsk 2020/WB-11558; ASFV/Khabarovsk 2022/WB-1650
16		**HK1**	MGF 360-14L-II (G -> A: 33161); B438L-II (C -> T: 98132); G1340L-IV (C -> T: 110920); NP419L-III (G -> A: 135380); D205R (C -> T: 138691); DP96R-II (C -> T: 185565)	HK/DP/2023/LFS-12807-34; HK/DP/2024/ST-00662-2 HK/DP/2023/ST-14219-23/1
17		**PG1**	MGF 505-3R-III (G -> T: 35925); MGF 505-5R-III (C -> T: 38327)	ASFV_PNG_2020_01; ASFV_PNG_2020_03; ASFV_PNG_2020_11; ASFV_PNG_2020_09; ASFV_PNG_2020_07; ASFV_PNG_2020_05; ASFV_PNG_2020_12; ASFV_PNG_2020_02; ASFV_PNG_2020_08; ASFV_PNG_2021_13; ASFV_PNG_2020_10
18		**CN3**	MGF 110-1L-II (G -> T: 7218); MGF 110-5L-6L-II (G -> C: 9571); QP383R-III (G -> A: 161687)	Pig/Heilongjiang/HRB1/2020; Pig/Hubei/628/2020
19		**CN1**	M1249L-II (C -> T: 80065)	wbBS01; CN/2019/InnerMongolia-AES01
20		**IN2**	MGF 360-11L-II (T -> C: 27619)	IND/AS/SD-02/2020; Nepal ASF 4
21		**PG2**	MGF 505-10R-III (C -> T: 47121)	ASFV_PNG_2023_15; ASFV_PNG_2023_14
22		**CN2**	O174L-III (G -> A: 129418)	GZ201801 2; China/2018/AnhuiXCGQ
23		**ID1**	E184L-II (G -> A: 163098)	A08200012-09-11; A02190909-4
24		**KR1**	MGF 360-10L-V (C -> G: 27183)	S-S-VR-413000-00008; Korea/Pig/Hongcheon/2021; Korea/Pig/Paju3/2019; Korea/Pig/Paju4/2019; Korea/YC1/2019; ASFV Korea/pig/Yeoncheon1/2019; Korea/HC224/2020; Korea/Pig/Hwacheon2/2020; Korea/Pig/Hwacheon1/2020; Korea/Pig/Yeuncheon2/2019; Korea/PC1432/2021; Korea/CW714/2020; S-S-VR-413000-00015; S-S-VR-413000-00002; Korea/Pig/Inje2/2021
25		**KR2**	285L-II (G -> A: 11277)	Korea/Pig/Gimpo1/2019; Korea/Pig/Ganghwa2/2019
26		**KR3**	MGF 360-1La-II (A -> G: 2329); MGF 360-4L-II (C -> G: 16649)	Korea/Pig/Goseong/2021; Korea/Pig/Inje1/2021; Korea/Pig/Yeongwol/2021
27		**CN4**	MGF 360-15R-IV (G -> A: 51024); F1055L-III (G -> A: 62016); I177L-II (A -> G: 174947)	HuB20; HB03A
28		**CN5**	MGF 505-9R-III (T -> C: 44902); F778R-III (C -> G: 59484); C717R-II (G -> T: 83184); G1340L-II (T -> A: 112638)	SY-1, SY-2
29		**PH1**	MGF 360-9L-II (G -> A: 25813); MGF 360-14L-II (C -> A: 32910)	ASFV2020-019-B; ASFV2020-013-B
30		**PH2**	EP153R-II (A -> C: 74090); I329L-II (G -> A: 173586)	A4; PAN20211A
31		**PH3**	L60L-II (C -> T: 5224); MGF 110-4L-II (G -> C: 8980); MGF 505-2R-II (G -> A: 35316); MGF 505-6R-II (G -> A: 40153 + G -> A: 40542); C717R-III (G -> A: 84245); G1340L-III (A -> G: 111935); CP2475L-II (G -> A: 119110)	NEC20230822001; MDR202311F; NEC20230929004B; NEC20230929004A; NEC20230726003

Note: The SNP position is aligned with the whole-genome sequence of the Georgia 2007/1 strain (NC_044959.2). The names of topotypes and genetic lineages are shown in bold. The background color corresponds to the color of the taxa on the phylogenetic tree (Figure 2).

**Table 2:** Comparative analysis of two sub-genotyping methods: distribution of isolates by SNPs into topotypes/genetic lineages based on whole genomes (current study) and determination of genetic groups (clusters) by polymorphism of 6 loci (classification by Gallardo C. et al., 2023 [38]).

№	Topotype/Genetic Lineage	CVR	IGR	O174L	K145R	MGF	ECO2	Genetic Group
1	CAU1/unclassified	I	I	I	I	I	I	1
2	CAU1/RU1	I	II	I	I	I	I	3
3	EU1/unclassified	I	II	I	I	I	I	3
4	EU1/RS1	I	II	I	I	I	I	3
5	EU1/RU6	I	II	I	I	I	I	3
6	EU1/LT1	I	II	I	I	I	I	3
7	EU1/RU7	I	II	I	I	I	I	3
8	EU1/RU8	I	II	I	I	I	I	3
9	EU1/IT1	I	II	I	I	I	I	3
10	EU1/IT2	I	II	I	I	I	I	3
11	EU2/unclassified	I	II	I	II	I	I	7
12	EU2/UA1	I	II	I	II	I	I	7
13	EU2/RU2	I	II	I	II/III	I	I	7/unclassified
14	EU2/PL1	I	II	II	II	I	I	6
15	ASIA1/unclassified	I	II/III	I	I	I	I	3/unclassified
16	ASIA1/IN1	I	II	I	I	I	I	3
17	ASIA1/RU3	I	II	I	I	I	I	3
18	ASIA1/RU4	XIII	II	I	I	I	I	unclassified
19	ASIA1/RU5	I	II	I	I	I	I	3
20	ASIA1/HK1	I	II	I	I	I	I	3
21	ASIA1/PG1	I	II	I	I	I	I	3
22	ASIA1/CN3	I	II	I	I	I	I	3
23	ASIA1/CN1	I	II	I	I	I	I	3
24	ASIA1/IN2	I	II	I	I	I	I	3
25	ASIA1/PG2	I	II	I	I	I	I	3
26	ASIA1/CN2	I	II	I	I	I	I	3
27	ASIA1/ID1	I	II	I	I	I	I	3
28	ASIA1/KR1	I	II	I	I	I	I	3
29	ASIA1/KR2	I	II	I	I	I	I	3
30	ASIA1/KR3	I	II	I	I	I	I	3
31	ASIA1/CN4	I	II	I	I	I	III	unclassified
32	ASIA1/CN5	I	II	I	I	I	I	3
33	ASIA1/PH1	I	II	I	I	I	I	3
34	ASIA1/PH2	I	II	I	I	I	I	3
35	ASIA1/PH3	I	II	I	I	I	I	3

Note: Designations of genetic variants and groups fully comply with the guidelines of Gallardo et al. [38]. In cases of new combinations of genetic variants, the group is designated as “unclassified”. Each classified genetic group is shown in a different color.

## Data Availability

The names of the isolates and accession number(s) can be found in the Supplementary Table S1.

## Supplementary Materials

The following supporting information can be downloaded at https://www.mdpi.com/article/10.3390/v18030346/s1. Table S1: The list of studied isolates with their NCBI GenBank accession numbers.
